# Use of complementary and alternative medicine by cancer patients in Colombia

**DOI:** 10.1186/s12906-023-04144-z

**Published:** 2023-09-14

**Authors:** Raúl Murillo, Nidia Pinto-Martínez, Norma Serrano, Claudia Uribe, Edgar Navarro, Jorge Duque, Andrés Yepes, Laura Olaya, Carolina Mariño, Olga Lucia Morales, Marcela Andrea Erazo-Muñoz, Diana Carolina Sánchez-Vega, Nicolás Martínez-Ramos

**Affiliations:** 1 Hospital Universitario San Ignacio, Diagonal 70B 214 Este Apt 104, Bogotá, Colombia; 2 Pontificia Universidad Javeriana, Bogotá, Colombia; 3 Hospital Internacional de Colombia-Fundación Cardiovascular, Bucaramanga, Colombia; 4 Universidad Autónoma de Bucaramanga, Bucaramanga, Colombia; 5 Universidad del Norte, Barranquilla, Colombia; 6 Oncólogos Asociados de Imbanaco, Cali, Colombia; 7 Clínica de Oncología Astorga, Medellín, Colombia; 8 Unidad Oncológica Surcolombiana, Neiva, Colombia; 9 Universidad de Valencia, Valencia, Spain; 10 Clinica Colsanitas, Bogotá, Colombia; 11 Clínica Universitaria Colombia, Bogotá, Colombia

**Keywords:** Complementary therapies, Integrative oncology, Neoplasms, Colombia

## Abstract

**Background:**

The use of complementary and alternative medicines (CAM) among cancer patients varies greatly. The available data suggest an increasing use of CAM over time and a higher prevalence in low- and middle-income countries. However, no reliable data are available from Latin America. Accordingly, we examined the prevalence of CAM use among cancer patients from six Colombian regions.

**Methods:**

We conducted a survey on cancer patients attending comprehensive cancer centres in six capital cities from different regions. The survey was designed based on a literature review and information gathered through focus groups on CAM terminology in Colombia. Independent random samples of patients from two comprehensive cancer centres in every city were obtained. Patients 18 years and older with a histopathological diagnosis of cancer undergoing active treatment were eligible. The prevalence of CAM use is reported as a percentage with the corresponding confidence interval. CAM types are reported by region. The sociodemographic and clinical characteristics of CAM users and non-users were compared using Chi square and *t* tests.

**Results:**

In total, 3117 patients were recruited. The average age 59.6 years old, and 62.8% were female. The prevalence of CAM use was 51.7%, and compared to non-users, CAM users were younger, more frequently women, affiliated with the health insurance plan for low-income populations and non-Catholic. We found no differences regarding the clinical stage or treatment modality, but CAM users reported more treatment-related side effects. The most frequent types of CAM were herbal products, specific foods and vitamins, and individually, soursop was the most frequently used product. Relevant variability between regions was observed regarding the prevalence and type of CAM used (range: 36.6% to 66.7%). The most frequent reason for using CAM was symptom management (30.5%), followed by curative purposes (19.5%).

**Conclusions:**

The prevalence of CAM use among cancer patients in Colombia is high in general, and variations between regions might be related to differences in cultural backgrounds and access to comprehensive cancer care. The most frequently used CAM products and practices have little scientific support, suggesting the need to enhance integrative oncology research in the country.

**Supplementary Information:**

The online version contains supplementary material available at 10.1186/s12906-023-04144-z.

## Introduction

Integrative oncology is defined as patient-centred, evidence-based and coordinated use of traditional complementary therapies with conventional oncology care, aimed at improving patients’ quality of life and clinical outcomes and to empower patient participation during the course of treatment [1]. Indeed, the use of complementary and alternative medicines (CAM) by cancer patients has been reported to be about 50%; however, there is high variability between reports, ranging from 10 to 90% [2], and in patients with advanced disease, the prevalence of CAM use can reach 100% [3].

Moreover, a review on CAM use in high-income countries found an increasing trend over time [4], while another review on cancer patients in low- and middle-income countries (LMIC) found slightly higher CAM use (54%), with only 26.7% of patients reporting complementary rather than alternative use [5]. Given the significant cultural influence of ancestral communities, the integration of CAM in the Americas region is an important issue, as indicated by the attempts to standardise and regulate traditional practices [6, 7]; however, bibliometric analyses reveal the scarce participation of Latin American countries in integrative oncology research [8, 9]. The systematic reviews mentioned above on prevalence of CAM use included only one study on paediatric patients from Guatemala, which had a small sample size obtained from a single centre [10]. We performed an additional search in Latin American databases (LILACS), finding only one multicentre study from Argentina, which reported an average CAM use of 90% among adult cancer patients [11].

In addition to its potential benefits for cancer care, the rise of integrative oncology is a response to the need to reduce the possibility of drug natural products interactions and to improve the safety of non-conventional practices [12]. Thus, a better understanding of the demand for integrative oncology requires not only knowledge about the prevalence of CAM use but also about the type of non-conventional products and practices that are being used and the evidence behind them. The cultural diversity in Latin America strongly influences health practices, and social disparities lead to extensive use of CAM [7, 13]. Further, the widespread use of the internet and social media has expanded non-conventional practices, including those with strong roots in ancestral communities but also useless and controversial treatments for cancer [14]. Therefore, gathering structured information about specific practices in Latin American countries would not only benefit integrative oncology practice but also provide relevant baseline information to foster integrative oncology research in order to improve the base of evidence for cancer care.

Colombia is a middle-income country with universal health insurance [15]. However, there are important disparities in access to cancer care in the country related to both socioeconomic conditions and geographic coverage [16, 17]. A report on CAM use among cancer patients at the cancer centre of the Colombian National Cancer Institute (NCI) indicated a prevalence of CAM use of about 70% [18]. Yet, the Colombian NCI receives patients mainly from Bogota, whereas the country has at least 21 comprehensive cancer centres distributed in six major regions with diverse cultural backgrounds [19]. Thus, this study aimed to determine the reasons for and prevalence of current CAM use among oncology patients in different Colombian regions and to identify specific practices in order to enhance the body of knowledge on this topic in Colombia and Latin America.

## Methods

We conducted a survey on cancer patients attending comprehensive cancer centres in Barranquilla (Atlantic coast), Bogota (Central high mountains), Bucaramanga (East medium mountains), Cali (Pacific region), Medellin (Coffee growing area) and Neiva (River valley). The protocol was centrally approved by the Ethical Committee at the Hospital Universitario San Ignacio-Pontificia Universidad Javeriana in Bogota, and we also obtained approval from review boards in all participating institutions.

In designing the survey, we gathered preliminary information about the terminology and the general lexicon around CAM in Colombia [20]. Through a series of structured focus groups with cancer patients and oncology care providers, we explored the reasons for and type of CAM use common to cancer patients as well as CAM practices known by oncology care providers. In addition to the areas explored in the focus groups, we collected data on the sociodemographic and clinical characteristics of participants.

A random sample of patients was selected between June 2019 and March 2023 in two comprehensive cancer centres per city, with the exception of Bucaramanga, where only one centre was included. Initially, we randomly selected workdays in outpatient services and carried out a sequential recruitment of patients in waiting rooms. However, due to the COVID-19 pandemic we combined the in-person recruitment with telephone surveys. For the latter, we randomly selected patients who had attended outpatient services during the previous week. The sequential recruitment of patients continued until we reached the estimated sample size. Trained personnel administered the survey (about 20 min long).

The eligibility criteria included age 18 years and older, a histopathological diagnosis of cancer and active treatment (systemic therapy, radiotherapy, surgery within the last four months, palliative care if not eligible for other treatment modalities). Patients of any clinical stage and with any type of cancer were eligible. Participation was voluntary, and we obtained verbal consent after explaining the objectives of the study and the content and estimated time required to complete the survey. While the patients could be accompanied by home care providers or relatives, only the patients could compete the survey items.

### Statistical analysis

An independent sample size was estimated for every city, assuming an infinite population and a prevalence of CAM use of 70% [18]. With 95% confidence, 5% precision and design effect 2, we expected to recruit 15 patients per workday, that is, 515 patients in every city (35 blocks), and 3150 patients in total.

We described the sociodemographic and clinical characteristics of participants using absolute and relative frequencies and stratified them by CAM users and non-users. The prevalence of CAM use is reported as a percentage with the corresponding confidence interval. To classify the type of CAM reported by the patients, we used the categories originally defined by the US National Center for Complementary and Alternative Medicine [21]. We did not find the updated classification operational for the purposes of this study. We then performed a cross-sectional analysis to explore the associations between the sociodemographic and clinical characteristics of participants and their use of CAM. A *p*-value < 0.05 was considered to indicate statistical significance.

## Results

In total, 3117 patients were recruited (average age 59.6 years, 62.8% female). The response rate was 91% and 72% for in person and telephone surveys, respectively. Most participants were Catholic, of low socioeconomic status and lived in urban areas (Table 1). The participants were evenly distributed by city as planned, but we also found an even distribution by health insurance plan for the whole sample. However, the percentage of CAM users who were part of the subsidised regimen was higher (insurance for low-income populations).

**Table 1:** Sociodemographic characteristics of participants

Characteristics	CAM users (*n* = 1610)		CAM non-users (*n* = 1507)		Total		*p* value
	n	%	n	%	n	%	
Age							
Mean (Range)	57.9(14.1)		61.4(15.1)		59.6 (18–98)		< 0.001
Sex							
Men	482	29.9	676	44.9	1158	37.2	< 0.001
Women	1128	70.1	831	55.1	1959	62.8	
Marital status							
Married	973	60.4	899	59.6	1872	60.1	0.358
Widow or Divorced	263	16.3	250	16.5	513	16.5	
Single	374	23.2	358	23.8	732	23.5	
Religion							
Catholic	1222	75.9	1217	80.8	2439	78.3	< 0.001
Other	331	20.5	217	14.4	548	17.5	
No religion	50	3.1	29	1.9	130	4.2	
Socioeconomic condition							
Low	1037	64.4	989	65.5	2026	65.0	0.762
Medium	509	31.6	462	30.7	971	31.2	
High	64	4.0	56	3.7	120	3.8	
Education							
None or primary school	520	32.3	644	42.7	1164	37.3	< 0.001
Secondary or technical	855	53.2	702	46.6	1557	50.0	
University or higher	235	14.6	161	10.7	396	12.7	
City-Region							
Barranquilla	262	16.3	263	17.5	525	16.9	< 0.001
Bogota	239	14.8	287	19.0	526	16.9	
Bucaramanga	352	21.9	176	11.7	528	16.9	
Cali	278	17.3	248	17.3	526	16.9	
Medellin	178	11.1	308	20.4	486	15.6	
Neiva	301	18.7	225	14.9	526	16.9	
Place of residence							
Urban	1243	77.2	1175	78.0	1243	77.6	0.639
Rural	367	22.8	332	22.0	699	22.4	
Health insurance							
Contributory	853	53.0	875	58.1	1728	55.4	0.042
Subsidized	680	42.2	577	38.3	1257	40.3	
Other	77	4.8	55	3.7	132	4.2	

The contributory health insurance regimen corresponds to people with payment capacity (payroll or independent contribution) whereas the subsidized system corresponds to people without payment capacity

The prevalence of CAM use was 51.7% (1610 patients), and CAM users tended to be younger and female. The percentage of Catholic patients was lower among CAM users than among non-users, and the level of education was higher among CAM users. Regarding the Colombian region, we found a higher percentage of users in Bucaramanga and Neiva and the lowest percentage of users in Medellin (Table 1).

**Fig. 1:**
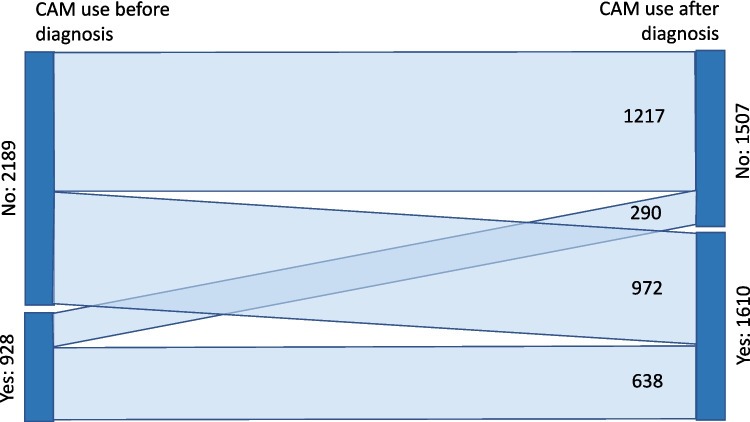
Prevalence of CAM use before and after the cancer diagnosis

Solid tumours were more frequent than haematological malignancies (Table 2), and among the former, breast and prostate cancers were the most prevalent. The percentage of haematological malignancies was lower among CAM users than among non-users (Table 2 and Supplementary file 1). Regarding the clinical stage of the disease at diagnosis, we found no differences between CAM users and non-users. In total, 46.7% reported a localised disease, and 50.2% reported that surgery was part of their treatment. A large majority (89.6%) were receiving systemic therapy. The percentage of patients reporting relevant treatment side-effects was higher among CAM users, and CAM use significantly increased after the diagnosis of cancer (29.8% vs 51.7% before and after, respectively, *p* < 0.001) (Fig. 1).

**Table 2:** Clinical characteristics of the study participants by CAM use condition

Characteristics	CAM users		CAM non-users		Total		
	n	%	n	%	n	%	*p*-value
Type of cancer^a^							
Hematological malignancies	159	9.9	237	15.7	396	12.7	< 0.001
Solid tumors	1451	90.1	1270	84.3	2721	87.3	
Clinical stage							
Localized	739	45.9	716	47.5	1455	46.7	0.422
Regional	301	18.7	248	16.5	549	17.6	
Distant	425	26.4	401	26.6	826	26.5	
Unknown	145	9.0	142	9.4	287	9.2	
Treatment^b^							
Systemic	1451	51.0	1342	51.4	2875	89.6	0.384
Radiotherapy	560	19.7	504	19.3	1064	34.1	
Surgery	820	28.8	746	28.6	1566	50.2	
Palliative care	13	0.45	19	0.72	32	1.0	
Treatment side effects							
Yes	1222	75.9	920	61.0	2142	68.7	< 0.001
No	388	24.1	587	39.0	975	31.3	

Clinical stage at diagnosis as reported by patients

^a^Detail data in the Supplementary file 1

^b^Every patient may have multimodal therapy

**Fig. 2:**
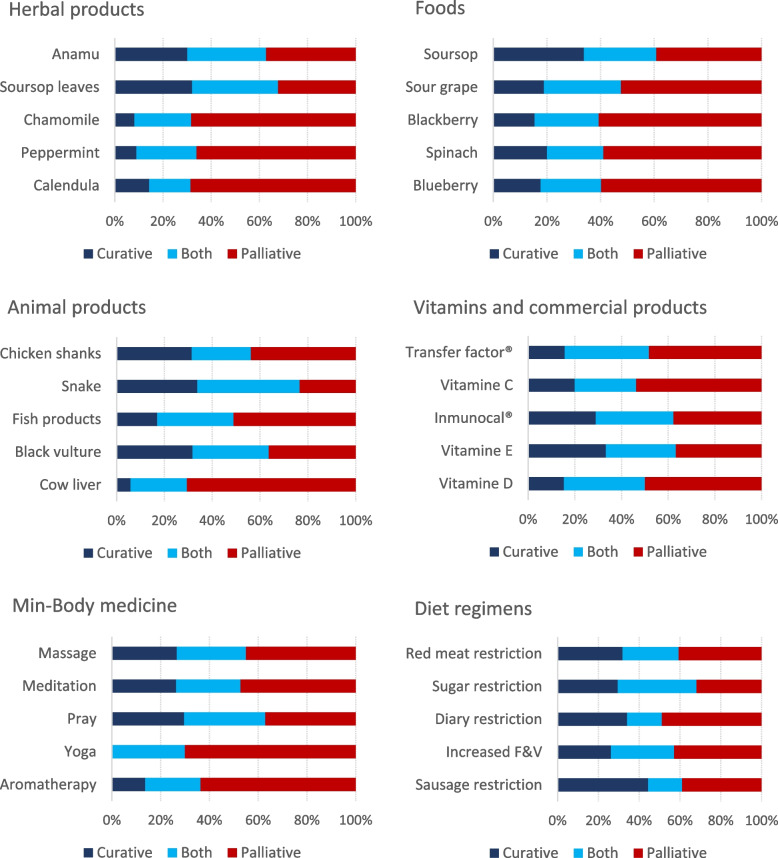
Reasons to use the most common products by CAM category in the study (percentage of self-reported users). F&V: Fruits and vegetables. To represent the percentages, variable values other than curative or palliative use have been ignored. Total numbers could be seen in the Supplementary file 2

The patients used multiple types of CAM simultaneously, but overall the most frequent types were herbal products, specific foods and vitamins (Table 3). Individually, soursop was the most frequently used product in all regions, followed by Transfer factors®, anamu, soursop leaves (herb) and sour grape (Fig. 2). Variability between regions was observed regarding the prevalence and type of CAM used. Bucaramanga had the highest percentage of CAM users compared to non-users (66.7%), followed by Neiva (57.2%) and Cali (52.9%). Meanwhile, Medellin had the lowest percentage of CAM users (36.6%). Additionally, the use of animal products was lower in Bogota, Cali and Medellin. Bogota had the lowest percentage of patients reporting diet regimens but also the highest percentage of patients using homeopathy.

**Table 3:** Type of CAM used by city/region

Type of CAM	Barranquilla *n* = 262 (%)	Bogota *n* = 239 (%)	Bucaramanga *n* = 352 (%)	Cali *n* = 278 (%)	Medellin *n* = 178 (%)	Neiva *n* = 301 (%)	All *n* = 1610 (%)
Natural products-based therapy							
Herbal products	155 (64.9)	104 (43.5)	184 (52.3)	164 (59.0)	90 (50.6)	187 (62.1)	887 (55.1)
Animal products	105 (40.1)	32 (13.4)	118 (33.5)	19 (6.8)	8 (4.5)	98 (32.6)	380 (23.6)
Nutrition							
Foods	189 (72.1)	114 (47.7)	221 (62.8)	142 (51.1)	76 (42.7)	139 (46.2)	881 (54.7)
Diet regimens	44 (16.8)	8 (3.3)	42 (11.9)	42 (15.1)	35 (19.7)	27 (9.0)	197 (12.2)
Vitamins	170 (64.9)	131 (54.8)	168 (47.7)	161 (57.9)	70 (39.3)	164 (54.5)	864 (53.7)
Whole medical systems							
Homeopathy	29 (11.1)	55 (23.0)	39 (11.1)	28(10.1)	19 (10.7)	14 (4.7)	184 (10.2)
Traditional practices	3 (1.1)	2 (0.8)	6 (1.7)	3 (1.1)	4 (2.2)	1 (0.3)	19 (1.2)
Energy medicine	14 (5.3)	20 (8.4)	9 (2.6)	27 (9.7)	21 (11.8)	7 (2.3)	98 (6.1)
Mind–Body medicine	54 (20.6)	39 (16.3)	51 (14.5)	39 (14.0)	21 (11.8)	44 (14.6)	248 (15.4)
Other	43 (16.4)	5 (2.1)	17 (4.8)	33 (11.9)	28 (15.7)	5 (1.7)	131 (8.1)

Percentages for column values. Traditional practices include Chinese medicine, ayurvedic medicine, traditional healers

Among CAM users, 30.5% of patients reported using CAM for symptom management or to improve their mood, while 19.5% use CAM with curative purposes either as an adjuvant for active treatment or to prevent cancer recurrence. Other reasons to use CAM included following a relative’s or friend’s recommendation (15.8%), following a medical indication (2.4%) and using CAM due to “its natural origin” (2%). Other patients reported using CAM as a desperate alternative or provided no specific reason for doing so. The most common products in the survey were used for curative purposes by more than 40% of CAM users (considering use for curative and palliative purposes simultaneously). Despite the low number of users, snake products were the products with the higher percentage of users with curative purposes (over 70%) (Fig. 2).

## Discussion

We found a 51.7% prevalence of CAM use among oncology patients in Colombia (range: 36.6% to 66.7% between Colombian regions). To the best of our knowledge, this is the largest multicentric survey on CAM use among oncology patients in Latin America and the only survey showing within-country variability of CAM products and practices.

A review found a CAM use prevalence of 54% in LMIC; however, no studies on adult patients from Latin America were included, and the large variability in the methods makes it difficult to understand the differences between the studies [5]. In an additional search, a report from Chile (single centre in Santiago) reported current and past CAM use together [22]; however, we found that the prevalence of CAM use significantly changes after a cancer diagnosis; thus, combining numbers before and after diagnosis artificially increases the prevalence of CAM use. We also found a report from Argentina (4 centres in Buenos Aires) indicating a 90% prevalence of CAM use, but it did not provide details on the methodology [23].

Like other Latin American countries, Colombia is a middle-income country with a large territory where populations with different cultural backgrounds and socioeconomic conditions coexist. Accordingly, our results show relevant differences between Colombian regions. The prevalence of animal product use was lower in the three largest cities in the country, possibly due to a higher level of urbanisation and a lower influence of empiric practices. However, it could be also related to better access to comprehensive cancer care and integrative oncology and, consequently, a better coordination of regular care with evidence-based and safe CAM practices. Yet, this does not explain the frequent use of commercial preparations lacking a solid base of evidence (Transfer factors® and Immunocal®); in this case, the active advertisement of these products, the abundant availability of information outside the health care setting, and inadequate communication about CAM use between patients and oncology care providers could explain the findings [20].

In fact, some of the most common products reported by the patients in this study have been the subject of in vitro and preclinical studies, although none of them has demonstrated anticarcinogenic effect in clinical trials. The most popular product, soursop (*Annona muricata*), is common in the Colombian diet and easy to find throughout the country. While *A. muricata* extracts have shown cytotoxic activity in vitro, some phytochemical compounds isolated from this fruit have also shown neurotoxic effects in vivo, thus warranting research to determine the clinical potential of the product [24]. Conversely, the product with the higher percentage of users with palliative purposes, *Calendula officinalis* (Fig. 2), has been evaluated in several clinical trials for managing radiation-induced dermatitis and mucositis [25]. Overall, mind–body medicine and nutrition are the most common practices in structured integrative oncology services and guidelines [26, 27], but we found only a 15.4% prevalence of use among cancer patients in Colombia. In summary, the lack of evidence for most of the products and the lower use of practices with better scientific support highlight the need to enhance integrative oncology services; nevertheless, this also represents an opportunity to develop integrative oncology research in the country and the region.

In addition to regional variations, we found that CAM users tended to be younger, female, non-Catholic and to have higher levels of education (Table 1). Although sociodemographic predictors of CAM vary significantly depending upon the setting, most studies from high-income countries have reported a similar profile regarding age, sex and education [2]. Reports from LMIC suggest a higher prevalence of CAM use due to the lower access to cancer care and higher out-of-pocket expenditure [28]. However, lower socioeconomic status was not associated with CAM use in our study, and previous reports from the Colombian NCI indicated that higher education was common among CAM users [18]. These results might be related with the universal health insurance coverage in Colombia; thus, while low access to health care is not a major determinant of CAM use, deficient access to integrative oncology could be, as it is related to an increased use of alternative information sources such as the internet, social networks and patient communities [20].

The clinical characteristics of the patients in the study did not differ significantly between users and non-users. The lower prevalence of haematological malignancies among users could be explained by a higher frequency of in-hospital care, which reduces the chance of CAM use. The higher prevalence of reported treatment-related side effects among CAM users clearly correlates with the higher percentage of palliative purposes of CAM use. There is no consistent association between clinical characteristics and CAM use among cancer patients in the available literature. Some studies from high-income countries have found a higher prevalence of early-stage disease among CAM users [2], whereas we found no association.

A review found no major differences between cancer patients and the general population regarding reasons to use CAM [29]; however, cancer patients more frequently reported expected benefits, autonomy, and the influence of social media as reasons for using CAM. These results might be interpreted as partially in agreement with our findings where symptom management (benefit) and curative purposes (internal locus of health control) were the most reported reasons for using CAM. A personal recommendation by friends or relatives was more common than social media in our study, but coherently, tradition has shown to be more influential in South America than in other world regions [29].

Our study has several limitations including non-response and recall biases. The sampling procedure, which involved a relatively small number of cancer centres, limits external validity; however, the sociodemographic characteristics of the sample closely represent the situation of the country regarding the health insurance plan, place of residence, education and socioeconomic conditions. The higher percentage of women in the survey is common for attendees to medical services willing to participate in surveys in general, but we also found differences in sex between CAM users and non-users. Further, a detailed investigation and in-depth analysis of the full list of products and their potential associations with particular clinical conditions is beyond the scope of the manuscript; thus, further analysis and studies are needed to better understand the determinants of CAM use in Colombia. Finally, we could not properly characterise homeopathy practices because the way in which patients refer to this medical modality is ambiguous, and they often refer to any kind of drug or product outside allopathic medicine as homeopathy.

## Conclusions

We deem our study relevant for Colombia and Latin America, as it is the largest survey conducted on CAM use in relation to oncology care in this world region. Specifically, it provides evidence regarding the within-country variability and diversity in CAM use for cancer care, highlighting the importance of structured integrative oncology services. Indeed, the prevalence of CAM use among cancer patients in Colombia is high and the observed variability in both the global prevalence and the type of CAM products and practices might be related to differences in cultural backgrounds and access to comprehensive cancer care, where products and practices with lower support on scientific evidence were less common in the biggest urban centres. However, the most frequently used CAM products and practices overall have little scientific support, suggesting the need to enhance integrative oncology services in the country and to improve integrative oncology research.

### Supplementary Information

**Additional file 1.** Most frequent cancer types as reported by the patients (All patients).

**Additional file 2.** Five most common products by CAM category in the study (number of patients).

## Data Availability

Datasets will be available upon request to Nicolas Martinez (martineznicolas@javeriana.edu.co).
