# Supplementary material for: Use of complementary and alternative medicine by cancer patients in Colombia
Source: BMC Complement Med Ther. 2023 Sep 14;23:321. doi: 10.1186/s12906-023-04144-z (PMC10500828; doi:10.1186/s12906-023-04144-z)
Supplement: Supplementary file 1 — Additional file 1. Most frequent cancer types as reported by the patients (All patients). [file 12906_2023_4144_MOESM1_ESM.docx]

Supplementary file 1. Most frequent cancer types as reported by the patients (All patients)

| ICD-10 | Cancer type | % |
| --- | --- | --- |
| C50 | Breast | 33% |
| C61 | Prostate | 10% |
| C18 | Colon | 6.6% |
| C16 | Stomach | 5.4% |
| C56 | Ovary | 4.0% |
| C90 | Multiple myeloma and malignant plasma cells | 3.4% |
| D | Neoplasms SAI | 3.1% |
| C34 | Bronchus and lung | 2.8% |
| C53 | Cervix uteri | 2.7% |
| C20 | Rectum | 2.2% |
| C92 | Myeloid leukemia SAI | 1.6% |
| Other |  | 25% |
