# Supplementary material for: Use of complementary and alternative medicine by cancer patients in Colombia
Source: BMC Complement Med Ther. 2023 Sep 14;23:321. doi: 10.1186/s12906-023-04144-z (PMC10500828; doi:10.1186/s12906-023-04144-z)
Supplement: Supplementary file 2 — Additional file 2. Five most common products by CAM category in the study (number of patients). [file 12906_2023_4144_MOESM2_ESM.pptx]

## Slide 1
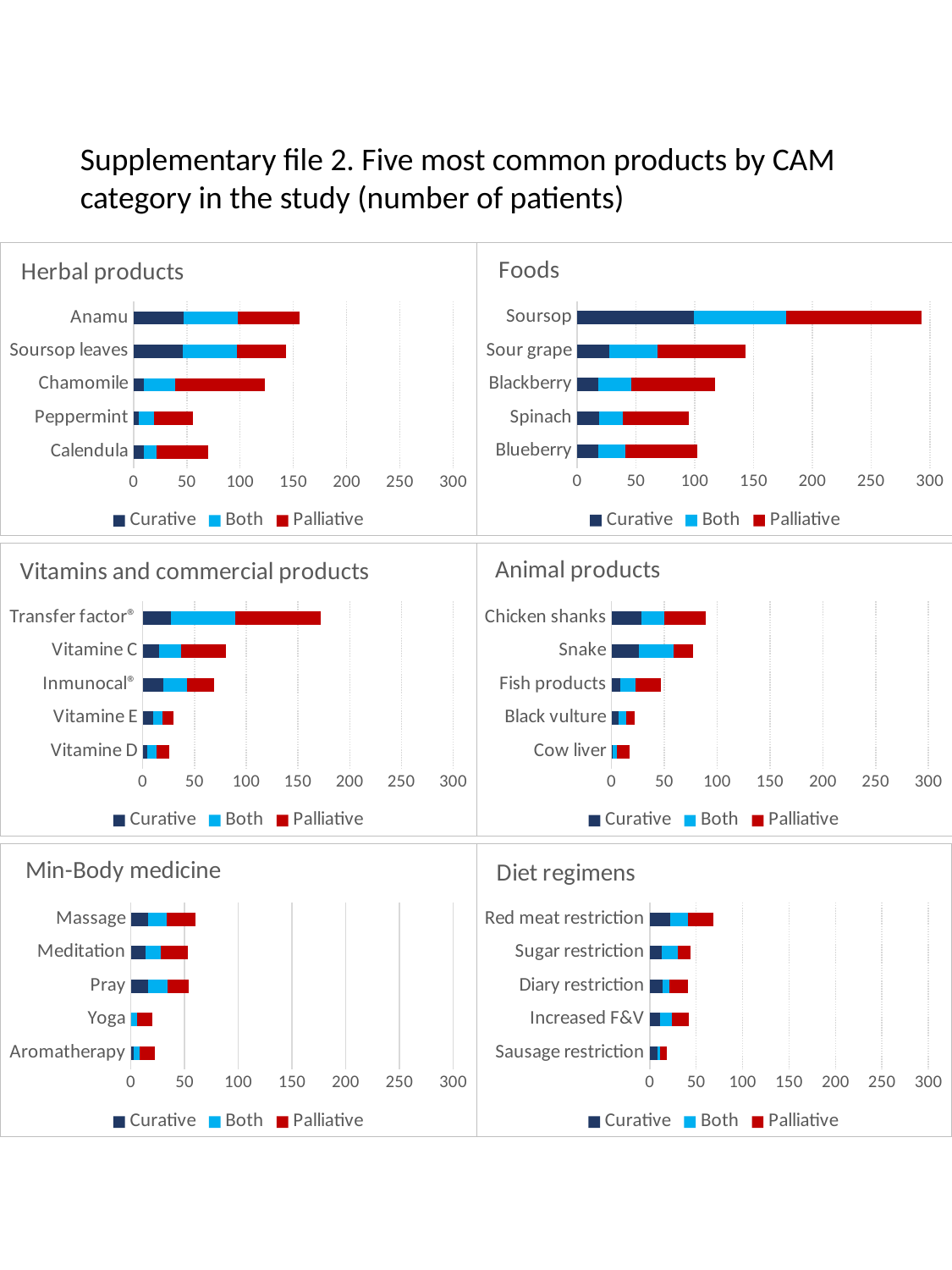

Supplementary file 2. Five most common products by CAM
category in the study (number of patients)
### Chart: Foods
| Category | Curative | Both | Palliative |
|---|---|---|---|
| Soursop | 99.0 | 79.0 | 115.0 |
| Sour grape | 27.0 | 41.0 | 75.0 |
| Blackberry | 18.0 | 28.0 | 71.0 |
| Spinach | 19.0 | 20.0 | 56.0 |
| Blueberry | 18.0 | 23.0 | 61.0 |
### Chart: Herbal products
| Category | Curative | Both | Palliative |
|---|---|---|---|
| Anamu | 47.0 | 51.0 | 58.0 |
| Soursop leaves | 46.0 | 51.0 | 46.0 |
| Chamomile | 10.0 | 29.0 | 84.0 |
| Peppermint | 5.0 | 14.0 | 37.0 |
| Calendula | 10.0 | 12.0 | 48.0 |
### Chart: Vitamins and commercial products
| Category | Curative | Both | Palliative |
|---|---|---|---|
| Transfer factor® | 27.0 | 62.0 | 83.0 |
| Vitamine C | 16.0 | 21.0 | 43.0 |
| Inmunocal® | 20.0 | 23.0 | 26.0 |
| Vitamine E | 10.0 | 9.0 | 11.0 |
| Vitamine D | 4.0 | 9.0 | 13.0 |
### Chart: Animal products
| Category | Curative | Both | Palliative |
|---|---|---|---|
| Chicken shanks | 28.0 | 22.0 | 39.0 |
| Snake | 26.0 | 33.0 | 18.0 |
| Fish products | 8.0 | 15.0 | 24.0 |
| Black vulture | 7.0 | 7.0 | 8.0 |
| Cow liver | 1.0 | 4.0 | 12.0 |
### Chart: Min-Body medicine
| Category | Curative | Both | Palliative |
|---|---|---|---|
| Massage | 16.0 | 17.0 | 27.0 |
| Meditation | 14.0 | 14.0 | 25.0 |
| Pray | 16.0 | 18.0 | 20.0 |
| Yoga | 0.0 | 6.0 | 14.0 |
| Aromatherapy | 3.0 | 5.0 | 14.0 |
### Chart: Diet regimens
| Category | Curative | Both | Palliative |
|---|---|---|---|
| Red meat restriction | 22.0 | 19.0 | 28.0 |
| Sugar restriction | 13.0 | 17.0 | 14.0 |
| Diary restriction | 14.0 | 7.0 | 20.0 |
| Increased F&V | 11.0 | 13.0 | 18.0 |
| Sausage restriction | 8.0 | 3.0 | 7.0 |
